# Paternal metabolic and cardiovascular programming of their offspring: A systematic scoping review

**DOI:** 10.1371/journal.pone.0244826

**Published:** 2020-12-31

**Authors:** Claudia Eberle, Michaela F. Kirchner, Raphaela Herden, Stefanie Stichling

**Affiliations:** Medicine with Specialization in Internal Medicine and General Medicine, Hochschule Fulda–University of Applied Sciences, Fulda, Germany; Hull York Medical School, UNITED KINGDOM

## Abstract

**Background:**

There is lots of evidence that maternal peri-gestational metabolic, genomic and environmental conditions are closely linked to metabolic and cardiovascular outcomes in their offspring later in life. Moreover, there is also lotsof evidence that underlining mechanisms, such as molecular as well as epigenetic changes may alter the *intrauterine* environment leading to cardio-metabolic diseases in their offspring postnatal. But, there is also increasing evidence that cardio-metabolic diseases may be closely linked to their paternal metabolic risk factors, such as obesity, Type 2 Diabetes and other risk factors.

**Objective:**

To analyse the evidence as well as specific risk factors of paternal trans-generational programming of cardio-metabolic diseases in their offspring.

**Methods:**

Within a systematic scoping review, we performed a literature search in MEDLINE (PubMed) and EMBASE databases in August 2020 considering original research articles (2000–2020) that examined the impact of paternal programming on metabolic and cardiovascular offspring health. Epidemiological, clinical and experimental studies as well as human and animal model studies were included.

**Results:**

From n = 3.199 citations, n = 66 eligible studies were included. We selected n = 45 epidemiological as well as clinical studies and n = 21 experimental studies. In brief, pre-conceptional paternal risk factors, such as obesity, own birth weight, high-fat and low-protein diet, undernutrition, diabetes mellitus, hyperglycaemia, advanced age, smoking as well as environmental chemical exposure affect clearly metabolic and cardiovascular health of their offspring later in life.

**Conclusions:**

There is emerging evidence that paternal risk factors, such as paternal obesity, diabetes mellitus, nutritional habits, advanced age and exposure to environmental chemicals or cigarette smoke, are clearly associated with adverse effects in metabolic and cardiovascular health in their offspring. Compared to maternal programming, pre-conceptional paternal factors might also have also a substantial effect in the sense of trans-generational programming of their offspring and need further research.

## Introduction

Evidence suggests that maternal metabolic, molecular genomic and environmental conditions might imprint metabolic and cardiovascular conditions in their offspring [1–6]. Hence, pathophysiological changes in intrauterine environment might “program” and predict those developments in the offspring early on [5]. In this context, trans-generational programming describes the perturbation at critical periods of development causing permanent lifelong alterations with irreversible consequences [1]. Hales and Barker’s “thrifty phenotype hypothesis” [7] is stating “that the epidemiological associations between poor fetal and infant growth and the subsequent development of type 2 diabetes mellitus and the metabolic syndrome result from the effects of poor nutrition in early life, which produces permanent changes in glucose-insulin metabolism” [7]. Further, the “Predictive Adaptive Responses Hypothesis” outlines that the fetus predicts the postnatal environment by “adapting” developmental processes *in utero* [1]. Whereas an altered fetal environment through *maternal* influences is very likely associated with the development of metabolic and cardiovascular diseases in later life, less is known about *paternal* factors influencing offspring health [8]. Hence, the “advanced fetal programming hypothesis” proposes that programming events related to paternal genes are affecting the fetal phenotype independently of the fetal genome [9]. According to that hypothesis, paternal environmental factors (e.g. body composition, endocrine function, nutritional habits, and age) might influence the offspring`s phenotype through epigenetic imprinting processes in sperm as the alterations in the paternal germline epigenome are passed on to the offspring [8–11]. Paternal under- and overnutrition can induce metabolic phenotypes in the offspring, and the induced phenotype can affect multiple generations [12]. The transfer of metabolic disease risk through male parentage implies an inheritable factor carried by sperm. Sperm-based transmission offers a comprehensible system for querying heritable epigenetic factors that influence the metabolism [12].

We conducted a systematic scoping review to summarize the updated evidence. We aimed at reviewing the existing evidence on whether and which paternal risk factors affect trans-generational programming and thus lead to adverse metabolic and cardiovascular outcomes in offspring. Then, we discuss the impact of paternal compared to maternal programming.

## Materials and methods

### Data sources, search and screening strategy

We performed a systematic scoping review to summarize the evidence available on the topic for the purpose of identifying potential paternal risk factors and different cardio-metabolic outcomes, outlining evidence gaps, reviewing various types of evidence and conveying the breadth of the topic [13]. This review combines systematic and scoping approaches using systematic, explicit methods to explore and describe a broad evidence base. We followed PRISMA for systematic reviews [14] (S1 Checklist) and Joanna Briggs Institute for systematic scoping reviews guidelines [13].

The electronic databases MEDLINE (PubMed) and EMBASE were searched in August 2020 using the following keywords as Medical Subject Headings and Embase Subject Headings terms and title/abstract terms:

- MEDLINE (PubMed): ((((((parental[Title/Abstract]) OR (paternal exposure[MeSH Terms])) OR (male*[Title/Abstract])) OR (father*[Title/Abstract])) OR (paternal*[Title/Abstract])) AND (((((preconception[Title/Abstract]) OR (prenatal[Title/Abstract])) OR (transgenerational[Title/Abstract])) OR ("fetal development"[MeSH Terms])) OR (programming*[Title/Abstract]))) AND ((cardiovascular disease[MeSH Terms]) OR (metabolic disease[MeSH Terms])) AND ((english[Filter] OR german[Filter]) AND (2000:2020[pdat]))
- EMBASE: ((((“paternal”:ti,ab) OR (“parental”:ti,ab) OR (“male”:ti,ab) OR (“father”:ti,ab) (“paternal exposure”/exp))) AND (“transgenerational”:ti,ab) OR (“programming”:ti,ab) OR “fetus development”/exp OR (“preconception”:ti,ab) AND (("cardiovascular disease"/exp OR ("metabolic disorder"/exp)))) AND (([embase]/lim AND ([english]/lim OR [german]/lim) AND [2000–2020]/py))

After database searching and elimination of duplicates, records were screened by title and abstract. Then, studies with full-text were screened and eligible publications were selected for inclusion. In addition, the reference lists of included studies were manually checked to identify further publications. No protocol has been published and two independent reviewers selected the publications.

### Eligibility criteria

Peer-reviewed studies with full-text including original data on the impact of paternal risk factors (e.g. obesity, diabetes mellitus, nutrition, smoking) on metabolic and cardiovascular programming in offspring were involved. We included studies published over the past 20 years (January 2000 until August 2020) in English and German. To encourage our scope, we included epidemiological (cross-sectional, cohort, case-control studies), clinical and experimental trials as well as animal model and human studies. We excluded reviews and meta-analyzes as well as editorials, conference abstracts, letter, notes, and comments.

### Data extraction and synthesis

We extracted the following information: author, publication date, study design, study population, paternal risk factors, cardio-metabolic outcomes (offspring), and main findings. P-values below 0.05 were considered statistically significant. Furthermore, P-values were taken unchanged from the papers. First, studies were synthesized according to epidemiological and clinical designs (1) and experimental designs (2). Second, they were organized regarding the paternal factors examined:

1. *Epidemiological and clinical studies*: BMI/obesity, birth weight, nutrition, diabetes mellitus, age, smoking and environmental chemical exposure.
2. *Experimental studies*: high fat diet and obesity, low protein diet, undernutrition, hyperglycemia, and environmental chemical exposure.

With the aim of providing an overview of the existing evidence regardless of quality, we have not carried out a formal quality assessment in compliance with the guidelines for systematic scoping reviews [13].

## Results

**Fig 1:**
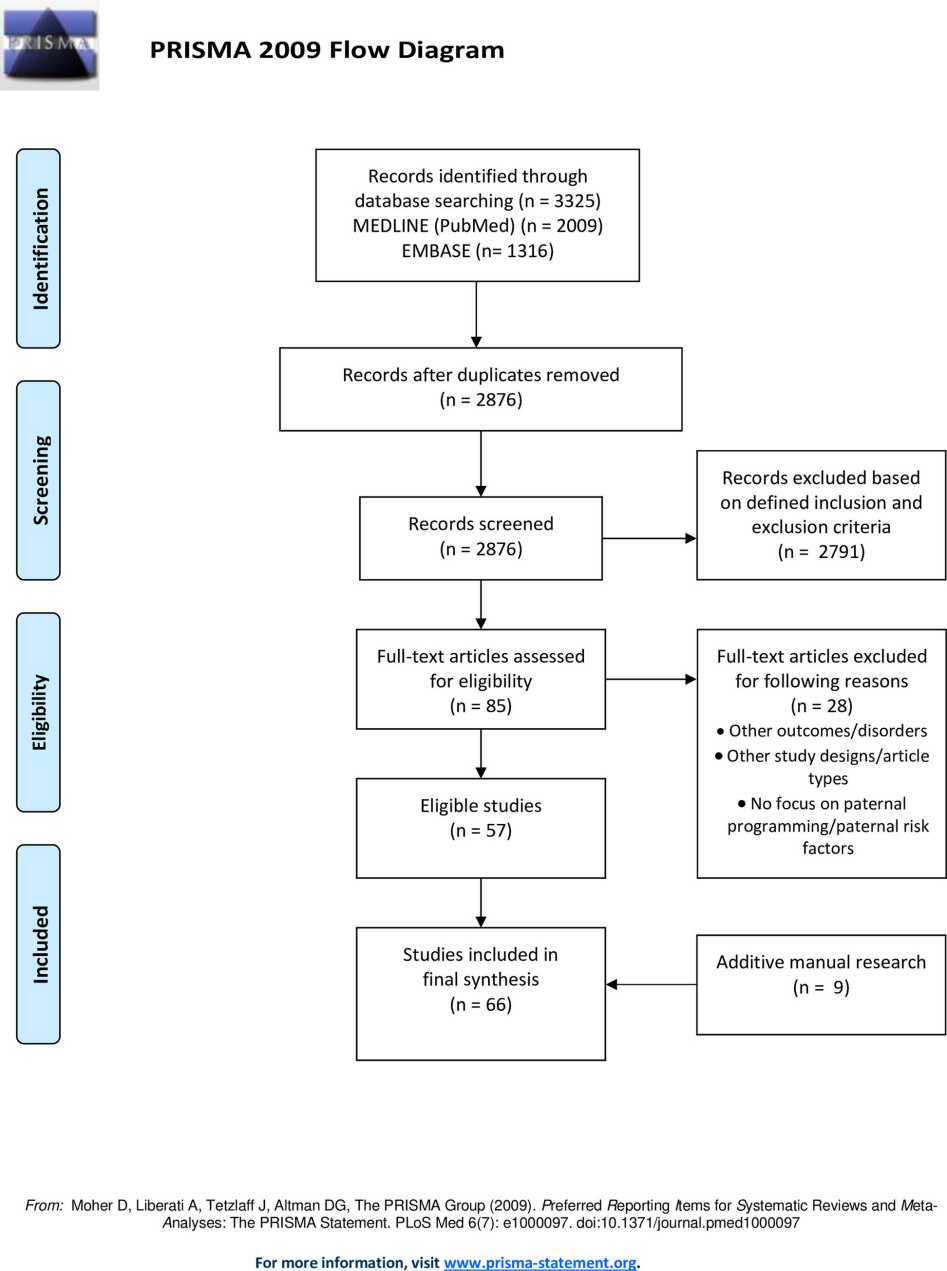
Flow diagram of the literature search and selection.

In total, n = 3.325 citations were identified. After the removal of the duplicates, n = 2.876 articles were screened by title and abstract, which led to the exclusion of n = 2.791 publications. We screened n = 85 studies with full-text for eligibility. After manual research of reference lists (n = 9 studies identified [15–21]), n = 66 studies were finally included in this systematic scoping review. We selected n = 45 epidemiological and clinical studies and n = 21 experimental studies. The search and selection process is shown in Fig 1. An overview of study characteristics is provided in S1 File.

Table 1 summarizes selected paternal programming effects and the distinction between female and male offspring.

**Table 1:** Paternal programming effects.

Paternal Risk Factor	Offspring	Main Findings	p-value
**BMI**	Female	Paternal BMI could not be associated with birth parameters of female offspring [22]	0.224
	Male	Paternal BMI was associated with birth weight in male offspring [22]	0.006
**Nutrition**	Female	Female offspring of high-fat diet fed fathers were heavier than offspring in control group, gained more weight and were insulin resistant [23]	<0.05
		Female offspring of obese fathers, induced by HFD, showed adiposity and insulin resistance; further, the females presented a reduced β-cell and islet area [24]	0.09
	Male	Male offspring of diet restricted fathers showed reduced fat mass, but an increased number of adipocytes, increased circulating lipids and free fatty acids; at 14 weeks insulin sensitivity was improved [25]	<0.05
**Diabetes mellitus**	Female	Positive association between a paternal history of diabetes and prediabetes in female offspring [26]	0.038
	Male	No significant association could be determined between a diabetic father and prediabetes in male offspring [26]	0.162
**Smoking**	Female	Early-onset paternal smoking was not significantly associated with female offspring BMI [21]	0.587
	Male	Paternal mid-childhood smoking is significantly associated with an increased BMI in boys at 9 years [21]	0.015
**Age**	Female	No significant influence of paternal age on female offspring regarding the function of the Renin-Angiotensin-Aldosterone-System or the pituitary-adrenal axis [22]	0.339
	Male	Paternal age has a significant influence on key hormone systems for cardiovascular diseases in male offspring (e.g. Renin-Angiotensin-Aldosterone-System or pituitary-adrenal axis) [22]	0.138

P-values below 0.05 were considered statistically significant.

### Paternal risk factors: Epidemiological and clinical studies

#### BMI and obesity

Chen et al. (2012) [22] (examining n = 899 newborns) demonstrated that the paternal BMI correlated with the birth parameters of male, but not female offspring concluding that the paternal BMI presents a risk factor for cardiovascular diseases in male adult offspring [22].

Sørensen et al. (2016) [20] reported a significant association between the pre-conceptional parental BMI and the BMI of the children at birth, 12 months and 7 years of age. The results showed that the association between anthropometrics of the mother and those of the offspring is stronger than between father and offspring. However, the differences diminished with advancing offspring age, becoming minor at 7 years of age [20]. In addition, Zalbahar et al. (2016) [19] found a positive association between paternal BMI and BMI and waist circumference (WC) in adult offspring.

Gaillard et al. (2014) [27] showed that paternal pre-conceptional BMI contributed to an adverse cardiometabolic profile in consecutive generations by showing a significant positive association between paternal and childhood BMI [27]. Offspring of obese fathers showed higher values of total body and abdominal fat mass as well as higher triglyceride, insulin and C-peptide levels in comparison to offspring from fathers with normal weight [27]. Similar findings were reported by McCarthy et al. (2015) [28] finding a significant positive association between paternal BMI and BMI, WC, and triglyceride levels of the offspring. Santos Ferreira et al. (2017) [16] and Labayen et al. (2010) [29] also found that both maternal and paternal BMI could be linked to an detrimental cardiometabolic profile in later life of the offspring.

A study by Magnus et al. (2018) [30] indicated that maternal and paternal pre-conceptional obesity was clearly associated with an increased risk of developing type 1 diabetes in the childhood. Within a prospective cohort study, Veena et al. (2013) [31] found that maternal and paternal obesity showed a positive correlation to obesity and fasting insulin concentrations in offspring [31]. Paternal obesity was associated with an increased childhood BMI and WC, a higher sum of skin folds, an elevated body fat percentage, and led to higher offspring fasting blood glucose levels and the development of an insulin resistance [31].

Soubry et al. (2013) [32] determined clear associations between pre-conceptional obesity and DNA methylation patterns of the imprinted Insulin-Like Growth Factor 2 (IGF2) in the offspring. They found a significant decrease in methylation at the differentially methylated regions (DMRs) of the IGF2 gene among newborns of obese fathers [32] and identified an inverse relationship between DNA methylation in offspring and paternal obesity. As low methylation at this DMR had already been linked with negative health outcomes, the authors assumed a pre-conceptional impact of paternal adiposity on the reprogramming of imprint marks during spermatogenesis [32].

#### Birth weight

McCowan et al. (2011) [33] (investigating n = 2002 couples) demonstrated that men who fathered SGA infants (= defined as a weight below the 10th percentile for the gestational age) showed actually lower birth weights than men who fathered non-SGA infants [33]. Therefore, birth size appears to be heritable through the paternal germ line. However, McCowan et al. could not confirm a strong inverse association between paternal birth weight and paternal obesity. Rather, the authors noted under consideration of varied populations that the relationship between birth weight and obesity in adulthood differed depending on gender and age [33]. Furthermore, Derraik et al. (2019) [34] reported that the likelihood of having an baby, which is large for gestational age (LGA), increased with a higher paternal birth weight and the father being tall [34].

#### Nutrition and nutritional habits

Kaati et al. (2002) [35] showed that if the father was exposed to a famine during his slow growth period, the offspring were protected against deaths caused by cardiovascular diseases. If the paternal grandfather experienced a famine during his slow growth period, the grandchildren tended to be safe from developing diabetes [35]. In contrast, if the paternal grandfather had access to a surfeit of food during their slow growth period, their grandchildren had a fourfold higher risk of dying from diabetes. Also, the food supply of the paternal grandfather could only be linked to the mortality of their grandsons, whereas the granddaughter`s mortality was associated with the paternal grandmother’s food supply [35].

According to Li et al. (2017) [36], at the exposure of famine, there was a significant increase in the risk of developing hyperglycemia and Type 2 Diabetes mellitus (T2DM) in adult offspring of the first generation, and the hyperglycemia risk increased significantly in the second generation, whereas a significantly increased risk of T2DM could not be confirmed [36].

#### Diabetes mellitus

Penesova et al. (2010) [37] demonstrated that fathers with an onset of diabetes before the age of 35 had leaner children, which further showed a decreased early insulin secretion [37]. Silva et al. (2017) [38] support this hypothesis as the authors were able to clearly link T2DM to an increased offspring BMI and elevated triglyceride levels. Wang et al. (2015) [39] also found a clearly positive association between parental diabetes and T2DM incidence in offspring. T2DM incidence in overweight subjects showed a stronger association with paternal than with maternal diabetes [39].

In the study by Praveen et al. (2012) [40], the offspring with a family history of T2DM showed clearly higher BMI values and higher plasma insulin, C-peptide and proinsulin levels as well as lower insulin sensitivity and β-cell compensation in the offspring [40]. There were no significant differences between offspring of diabetic mothers and those of diabetic fathers [40].

Furthermore, Linares Segovia et al. (2012) [41] reported the highest BMI values of the offspring in families, in which both parents were diabetic, whereas the lowest glucose and total cholesterol levels were determined in offspring of healthy parents. Almari et al. (2018) [26] outlined that parental history of diabetes did not clearly increase the prevalence of prediabetes, but parental history of diabetes in addition to obesity in offspring [26]. Maternal diabetes was solely related to prediabetes among male offspring [26]. Shields et al. (2006) [42] demonstrated that paternal insulin resistance influenced the umbilical cord insulin concentrations in a way which was clearly contributing to the development of a fetal insulin resistance, independent of maternal factors [42].

In addition, Myklestad et al. (2012) [43] (n = 14,000 families) found a link between low birth weight in offspring and an increased cardiovascular risk among fathers, as well as a relation between low offspring birth weight and unfavorable glucose levels, increased blood pressure and high BMI values among fathers [43]. Hillman et al. (2013) [44] showed that fathers of offspring with a fetal growth-restriction were more likely to be insulin resistant, hypertensive and obese compared to fathers of normal grown offspring [44]. Furthermore, Moss et al. (2015) [45], Hyppönen et al. (2003) [46] and Veena et al. (2006) [47], showed that paternal diabetes was obviously associated with low offspring birth weight. Lindsay at al. (2000) [48] determined that the development of paternal diabetes can be predicted by the offspring’s birth weight. Thereby, the highest diabetes risk was identified in fathers of children in the lowest quintile of birth weight. They found a distinct association between low birth weight and an elevated diabetes risk in the offspring itself. Lauenborg et al. (2011) [49] could demonstrate that adult offspring with low birth weight and diabetic fathers showed decreased insulin sensitivity as well as increased plasma glucose levels in the state of fasting and after oral glucose load [49].

#### Age

Advanced paternal age has been associated with a higher risk of spontaneous abortions, stillbirth, preterm birth as well as with congenital malformations, childhood cancer, epilepsy, autism and schizophrenia in offspring [50]. Zhu et al. (2008) identified a U-shaped association between paternal age and mortality rates of children [50]. Urhoj et al. (2014) [51] reported that the risk of an under-five mortality increased significantly if the father was older than 40 years at the time of child birth by increased likelihood of dying from congenital malformations or malignancies [51]. In addition, Zhu (2005) [52] found that the prevalence of malformations of extremities and syndromes of multiple systems (e.g. Down´s syndrome) increased with advancing paternal age [52]. Althrough Su et al. (2015) [53] showed no overall association between the father’s age and heart defects in offspring, advanced paternal age could be linked to an elevated prevalence of patent ductus arteriosus in the offspring, which is a subtype of congenital heart defects [53]. Khandwala et al. (2018) [54] linked advanced paternal age to an increased risk of premature birth and a low offspring birth weight.

#### Smoking and environmental chemical exposure

Marczylo et al. (2012) [55] were able to show that cigarette smoke induced differential microRNA expression in the spermatozoa of smokers (compared to non-smokers). These altered microRNAs mediate pathways were essential for sperm and embryo development [55].

Pembrey et al. (2006) [42] focused on cigarette-induced transgenerational effects on offspring growth. Thereby, the authors found out that there is a transgenerational effect of paternal mid-childhood smoking on offspring BMI at 9 years. However, this effect was only observed in boys (see Table 1). Based on their observation that exposure in the slow growth period can lead to a transgenerational effect, Prembrey et al. proved the existence of a sex-specific, male-line transgenerational response system in humans, which is presumably mediated by the gonosomes. Further, the authors assumed that this male transgenerational response is carried by the sperm´s chromosomes e.g. through viruses, prions, RNA molecules or responsive DNA sequences [42].

De Jonge et al. (2013) [56] reported that paternal smoking of 15 cigarettes per day or more was associated with an increased risk of hypertension in adult offspring. Further, Dior et al. (2014) [57] displayed a positive association between maternal and paternal smoking and offspring weight, height and BMI at the age of 17 and a negative association with pulse rates. Similar findings were found at the age of 32 if at least one parent was smoking [57].

Golding et al. (2019) [56] demonstrated that regular paternal cigarette smoking before the age of 11 was strongly associated with an elevated fat mass in adulthood of the respective children [58]. However, these findings do not agree with the study results of Carslake et al. (2016) [59] which found no clear association between paternal early-onset smoking (before the age of 11) and higher BMI values in offspring [59]. Instead, another study by Dougan et al. (2016) [60] could show that grand-paternal smoking during pregnancy of the grandmother was associated with a higher risk for granddaughters at the age of 12 to be overweight or obese. A link between grand-paternal smoking and the BMI of the grandson was not established [60]. Deng et al. (2013) [61] and Cresci et al. (2011) [62] reported that paternal smoking was associated with conotruncal heart defects.

Beside cigarette smoke, there are various other chemicals, which also represent transgenerational risk factors for offspring health. In this context frequently mentioned chemicals are persistent organic pollutants (e.g. dioxins or insecticides), which belong to the group of developmental toxicants [63, 64]. Robledo et al. (2015) [63] were able to show that birth size and weight of the offspring was affected by the pre-conceptional paternal exposure to persistent organic pollutants. In contrast, Lawson et al. (2004) [64] did not found an association between paternal exposure to dioxins and adverse pregnancy outcomes. Other studies, which focused on occupations of fathers that involve contact with toxic substances, also provide conflicting and no clear results regarding the effects on offspring health [65, 66].

### Paternal risk factors: Experimental studies

#### High-fat diet and obesity

Ng et al. (2010) [24] investigated the effect of an induced high-fat-diet on F1 female offspring. They hypothesized that an intergenerational transmission of obesity and metabolic diseases can be initiated by the father through exposure to a high-fat-diet. The authors analyzed male rats (high-fat-diet or control diet) with females (control diet). The high-fat-diet fed male rats exhibited increased body weight, energy intake, adiposity, plasma leptin and liver mass, as well glucose intolerance and insulin resistance compared to rats on control diet, and their female litter showed adiposity and insulin resistance similar according their fathers [24]. Female progeny presented increased blood glucose, reduced insulin secretion as well as reduced β-cell and islet area [24] (see Table 1).

Masuyama et al. (2016) [67] demonstrated that offspring of high-fat diet fed male rates showed a metabolic syndrome-like phenomena, which includes weight and fat gain, glucose intolerance as well as elevated total triglyceride, decreased adiponectin, and increased leptin levels. This phenomena could be observed across two generations [67]. In addition, Ornellas et al [68] showed an impaired glucose metabolism and lipogenesis (without an influence on beta-oxidation) and enhanced hepatic steatosis in male high-fat diet fed mice.

In accordance with Ng et al. (2014) [69], McPherson et al. (2015) [23] outlined that paternal obesity in mice, which was induced by a high-fat diet prior to conception, caused insulin resistance and increased the accumulation of adipose tissue in their female offspring. Short-term diet and exercise interventions in fathers improved the metabolic health of the female offspring [23] (see Table 1).

Fullston et al. (2013) [70] could demonstrate that paternal exposure to a high-fat diet in mice, which caused obesity, induced a specific transgenerational phenotypic constellation of impaired glucose tolerance, insulin resistance in both male and female offspring [70]. Another study by Fullston et al. (2015) showed that both paternal obesity at conception and a consumption of a high-fat diet by an individual animal caused the development of a metabolic syndrome and subfertility.

A growth deficit in mate rat offspring of high-fat diet fed fathers, which led to a body weight reduction of 10% at the age of six months and which resulted in smaller fat pads and less muscle mass was detected by Lecomte et al. (2017) [71]. Krout et al. (2018) [70] showed that paternal exercise before conception can reduce the offspring’s risk of developing T2DM, which was induced by the fathers high-fat diet, assuming epigenetic alterations in sperm DNA. Further, Consitt et al. (2018) noted that a pre-conceptional paternal high-fat diet enhances skeletal muscle insulin sensitivity as well as whole-body insulin sensitivity in the early life of the offspring [72]. Offspring of high-fat diet fed fathers were more susceptible to gain body fat in the early stage of adulthood [72]. Further, Fullston et al. [73] indicated a high fat diet-induced paternal initiation of subfertility in offspring of two generations of mice.

#### Low protein diet

In the study by Watkins and Sinclair (2014) [74], adult offspring of low protein diet-fed male mice developed a significantly impaired cardiovascular and metabolic homeostasis, vascular dysfunction, impaired glucose tolerance as well as elevated adiposity in adulthood [74].

Paternal low protein diet in mice especially affected the signaling pathways of the lipid metabolism according to Carone et al. (2010) [75]. A significant increase in the relative concentration of saturated cardiolipins, saturated free fatty acids and saturated and monounsaturated triacyl-glycerides was observed in the progeny [75].

#### Undernutrition

Referring to Anderson et al. (2006) [76], paternal food deprivation in male mice caused a consistent decrease in average serum glucose and changes in corticosterone and insulin-like growth factor 1 in both male and female offspring [76].

McPherson et al. (2016) [25] noted that undernutrition in mice led to dyslipidemia, accumulation of adipose tissue, an altered expression of pancreatic genes, and reduced weight in male and female offspring [25] (see Table 1). In addition, vitamin and antioxidant supplements given to undernourished fathers normalized offspring weight and growth [25].

#### Hyperglycemia

Grasemann et al. (2012) [77] indicated in mice, that metabolic parameters were affected more by maternal than paternal hyperglycemia, and the skeletal development (changes in bone mineral content, trabecular structure, and cortical bone properties) was more affected by paternal hyperglycemia [77]. According to Shi et al. (2017) [78], the adult offspring of hyperglycemic male rats showed a significant weight gain, larger body size and an extensive expansion of adipose tissue resulting in obesity of the offspring. Glucose intolerance, reduced insulin sensitivity and impaired hypothalamic leptin signaling was identified in male offspring [78]. Similar conclusions were drawn by Li et al. (2019) [79] showing an increased liver weight, elevated plasma total cholesterol, triglyceride as well LDL levels as an accumulation of triglycerides in the liver in adult rat offspring of hyperglycemic fathers. Furthermore, the authors detected epigenetic alterations affecting the PPAR-alpha promoter in the liver of the offspring [79].

Wei et al. (2014) [80] determined that paternal prediabetes affected the overall methylation patterns in pancreatic islets of offspring and in sperm of the father [80].

#### Environmental chemical exposures

Lane et al. (2014) [81] observed in mice that an increased level of reactive oxygen species (ROS) in sperm and/or seminal fluid influences offspring health outcome in a negative way by poorer embryo development and reduced fetal growth. The ROS concentration was significantly increased in relation to cancer, smoking, obesity, chemical exposure and ageing [81]. Daughters from exposed fathers were smaller, developed a glucose intolerance and exhibited an increased adipose tissue accumulation [81]. Referring to McPherson et al. (2016), ROS mediated sperm DNA lesions could be reduced by vitamin and antioxidant supplementation, which increased the sperm health and correlated negatively with the postnatal growth of the male offspring [25].

Genotoxic agent benzo[a]pyrene (B[a]P) is a component of diesel emissions, tobacco smoke and smoked food products [82]. Godschalk et al. (2018) [82] observed a down-regulation of mitochondrial proteins in offspring of B[a]P exposed mice, and specially in male offspring an additional reduction of mitochondrial DNA copies could be identified [82].

## Discussion

### Principal findings

**Fig 2:**
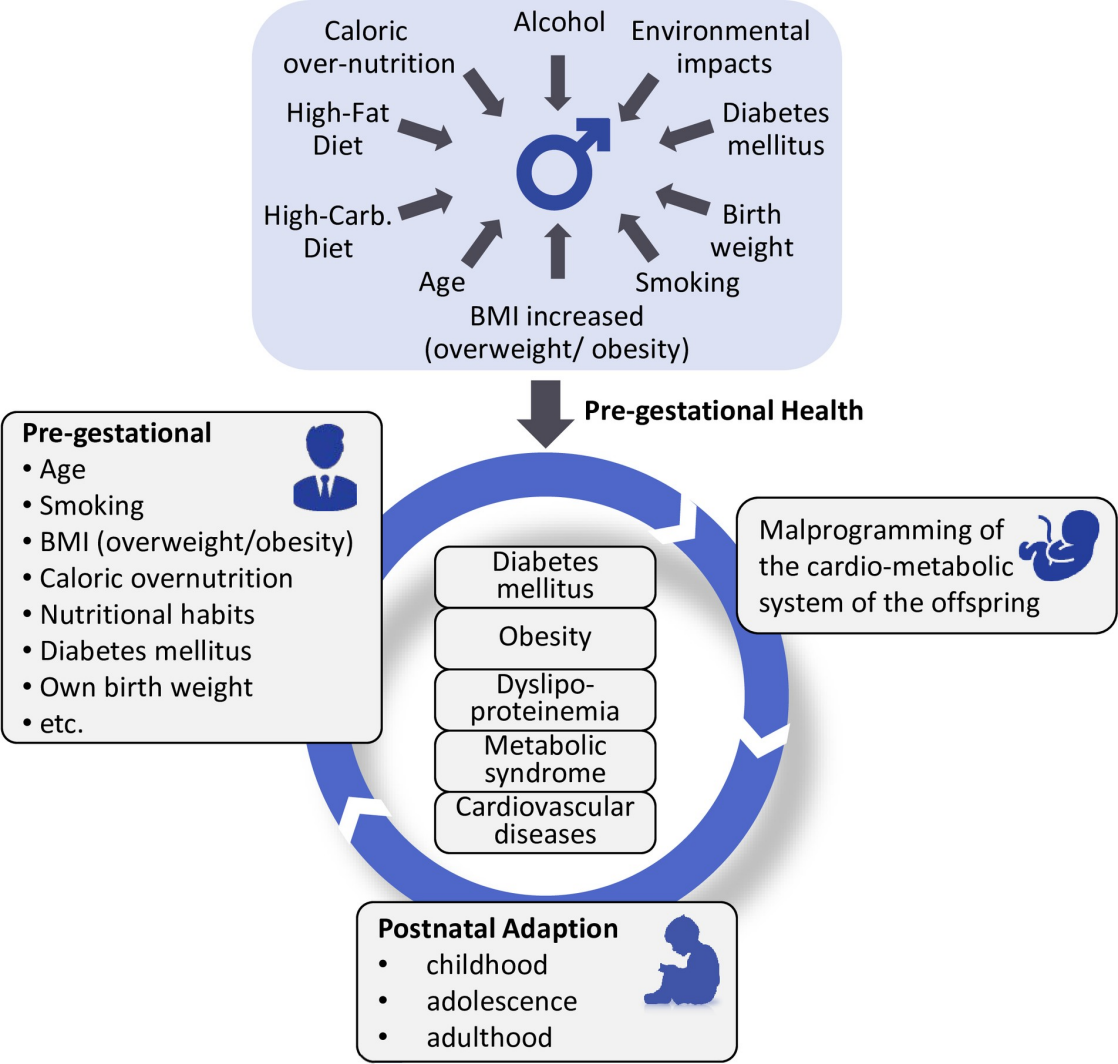
Life cycle of paternal programming.

This systematic scoping review identified possible paternal risk factors affecting trans-generational cardio-metabolic programming such as obesity, birth weight, high-fat and low-protein diet, undernutrition, diabetes mellitus, hyperglycaemia, advanced age, smoking as well as environmental chemical exposure (Fig 2).

As one of the most common adverse life style factors, paternal obesity could be pointed out as initiator of changes in sperm epigenetics, such as alterations in sperm DNA methylation and acetylation patterns [73]. Paternal obesity can lead to metabolic disturbances and changes in the transcriptome of genes in pathways regulating cellular response to stress, cell death and cell growth in adipose tissue of consecutive generations [23]. Further, male pre-conceptional obesity is able to reduce fetal growth, causes cardiovascular diseases and might be responsible for alterations in the glucose metabolism of the offspring. Also, an increased risk of developing obesity and insulin resistance in the offspring’s later life is associated with a high paternal BMI [16, 22, 27, 28]. Metabolic impairments in offspring are often associated with the paternal diet (high fat or low protein) and his nutrition status. For example, a high-fat diet of fathers in the time of conception is related to an impaired glucose metabolism, insulin resistance, weight gain, elevated triglyceride and increased leptin levels in the following generation [24, 67, 68]. A diet based on foods with low protein can also lead to an impairment of the cardiovascular and metabolic homeostasis [74]. In addition, the nutrition status of the father plays an important role in the context of metabolic offspring health. So, a prenatal exposure to a famine might increase the offspring’s risk of developing diabetes in later life [36]. In conclusion, obesity and the nutrition status of the father might play an important role in the context of metabolic offspring health [36].

Paternal diabetes was associated with a higher probability of developing diabetes in offspring [39]. Furthermore, children with diabetic fathers displayed an impaired insulin sensitivity and higher BMI values than children of non-diabetic fathers [40, 41]. In addition, advanced paternal age has been shown to induce DNA damage and de novo mutations. These factors entail considerable health risks for the offspring of aged fathers, e.g. low birthweight, preterm birth, congenital malformations, mental disorders or an increased overall mortality risk under the age of five [50–53].

Furthermore, smoking induces alterations in spermatozoa microRNA which are essential for sperm and embryo development [55]. Tobacco smoke initiates cardiovascular diseases, cancer, chronic lung diseases and increases the risk of adult-onset hypertension and overweight in offspring [56, 58, 61, 83]. In addition, the indirect harm done by maternal passive smoking has to be taken into account [83]. At last, the results of paternal exposure to different chemicals, which may trigger DNA mutations through increased ROS level in sperm, have been summarized. Amongst others, the exposure of paternal sperm to toxic chemicals led to poorer embryo development and reduced fetal growth [63, 81]. Further, negative alterations in the mitochondrial metabolism could be detected [82].

Our findings are in line with other reviews. Bodden et al. (2019) [84] reported that paternal obesity as a result of an excessive consumption of high calorie foods, leading to metabolic and neurobiological changes, can predict adverse offspring health outcomes. In addition, Sharp and Lawlor (2019) [85] linked paternal factors (e.g. paternal age, environmental exposures, high-fat-diet-induced obesity) to offspring development of obesity and type 2 diabetes. Likewise, Li et al. (2016) [86] indicated that paternal under and overnutrition, environmental toxin exposure, paternal diabetes, and grandfather's nutritional status can program cardio-metabolic diseases in offspring via germ cell-mediated transmission. Further, Campbell and Mcpherson (2019) [87] found that increased paternal BMI clearly affected pregnancy and offspring health outcome, for example leading to an increased BMI in childhood.

Furthermore, epigenetic mechanisms linking paternal well-being to offspring health have been analyzed. Watkins et al. [88] highlighted the role of the seminal plasma for offspring programming independent from that of the sperm. Chen et al. (2016) [89] indicated that tsRNA isolated from sperm from obese male mice recapitulates the paternal programming of offspring ill-health when compared to interact sperm. Lambrot et al. (2013) [90] found that epigenetic transmission may contain sperm histone H3 methylation or DNA methylation. Adequate paternal dietary folate is substantial for offspring health. In addition, according to Chan et al. (2020) [91], extracellular vesicles as a normal process in sperm maturation can perform roles in intergenerational transmission of paternal environmental experience.

Furthermore, limitations might have an impact on our findings. To the best of our knowledge and considering our inclusion and exclusion criteria, we involved all eligible studies. The publications were heterogeneous regarding study population, and evidence on some individual risk factors is limited. In some sections, such as the risk factor nutrition, we could only identify a few studies.

#### Maternal vs. paternal programming

The transmission of parental phenotypes to the offspring can be influenced by different environmental factors that induce epigenetic changes in oocytes and sperm [80, 92]. Epigenetic changes include variations in levels of DNA methylation, histone modification and the regulation of non-coding RNAs [23, 80]. Male individuals can affect offspring health through the quality of their sperm [80]. In case of female individuals, different environmental and lifestyle factors can shift epigenetic conditions leading to an adverse intrauterine environment. Lifestyle factors affect epigenetic modifications in genes of oocytes and decrease the oocytes’ quality. Alterations of the maternal intrauterine environment and reduction of the quality of oocytes may result in impaired fetal growth or developmental defects [93, 94].

A high maternal pre-conceptional BMI is known to be an important risk factor for adiposity, insulin resistance, impaired glucose tolerance and cardio-metabolic disease risk in the offspring [95, 96]. Other than maternal obesity, the father’s obesity seems to greatly affect the amount and distribution of bodyfat as well as adipokine levels in offspring of the next two generations [20, 27, 28]. Especially female offspring showed an impaired glucose-insulin homeostasis, which was transmitted via paternal lineage [23]. In male offspring, paternal obesity was associated with decreased fertility [73].

Both maternal and paternal diet high in fat and sugar seem to lead to impaired glucose and insulin metabolism, higher risk developing T2DM, adiposity, hypertension and hepatic disease in the offspring’s later life [67, 97].

Maternal diabetes was strongly associated with an increased birth weight and elevated diabetes risk in the offspring [47] and maternal transmission of T2DM is threefold higher than paternal transmission [98]. Paternal diabetes was clearly linked to low birth weight in the offspring, lower gestational age [47], influencing glucose and insulin levels, and increased risk of T2DM in adulthood [80].

*In utero* exposure to maternal smoking was linked to obesity, T2DM and cardiovascular diseases in adult offspring [57]. In comparison with maternal smoking, pre-conceptional paternal smoking as well as paternal smoking during pregnancy were associated with an increased risk of congenital malformations and heart defects in the offspring [61, 62]. Studies also showed an association between paternal smoking and an elevated risk of hypertension in later offspring life as well as increased BMI [21].

Furthermore, advanced maternal age was linked to an elevated risk of pregnancy complications, preterm birth and cardiovascular diseases in adult offspring [99]. Velazquez et al. (2016) could demonstrate that offspring from aged mice was prone to hypertension and showed higher weight gain in post-natal life than offspring of young female mice [100]. One explanation for these adverse pregnancy outcomes might be a decreased egg quality and an altered uterine environment of the mother [101]. In comparison with maternal age, there was also an obvious association between aged fathers and an increased risk for stillbirth, preterm birth, congenital malformations and offspring death [50, 51, 53, 54].

In general, comparable to maternal programming, paternal factors also have a substantial effect on trans-generational programming leading to adverse metabolic and cardiovascular outcomes in their offspring.

## Conclusions

In total, the evidence from several epidemiological, clinical, and experimental human and animal model studies indicates that paternal risk factors such as obesity, high-fat and low-protein diet, undernutrition, diabetes mellitus, hyperglycemia, advanced age, smoking as well as environmental chemical exposure might affect offspring health leading to adverse metabolic and cardiovascular outcomes (Fig 2). Comparable to maternal programming, pre-conceptional paternal factors might also have a substantial “programming” effect. Additional research on paternal risk factors, the underlying physiological mechanism of paternal programming, and the trans-generational inheritance is needed.

Considering our findings, an appropriate pre-conception care including medical, behavioural and social health interventions is very important to reduce the risk of epigenetic disorders and negative environmental exposures in order to improve offspring health as well as the parental health status [102]. Preventive and educational approaches clearly include both, mothers and fathers (to be), to reduce adverse health outcomes in their offspring caused by modifiable lifestyle and environmental risk factors effectively.

## Supporting information

S1 Checklist PRISMA checklist. (DOC)

S1 File (DOCX)

S2 File (DOCX)
