# Supplementary material for: Paternal metabolic and cardiovascular programming of their offspring: A systematic scoping review
Source: PLoS One. 2020 Dec 31;15(12):e0244826. doi: 10.1371/journal.pone.0244826 (PMC7775047; doi:10.1371/journal.pone.0244826)
Supplement: S2 File — (DOCX) [file pone.0244826.s003.docx]

S2 File: Study characteristics.

| **Authors** | **Year** | **Parental risk factor** | **Outcomes Offspring** | **Main findings** |
| --- | --- | --- | --- | --- |
| **Epidemiological and clinical studies** | | | | |
| Chen et al. | 2012 | BMI | cardiovasculcar diseases, fetal growth | paternal BMI correlated with the birth parameters of male, but not female offspring;  paternal BMI presents a risk factor for cardiovascular diseases in male adult offspring |
| Sørensen et al. | 2016 | BMI | BMI | significant association between the pre-conceptional parental BMI and the BMI of the children at birth, 12 months and 7 years of age |
| Zalbahar et al. | 2016 | BMI | BMI, waist circumference (WC) | positive association between paternal BMI and BMI and waist circumference (WC) in adult offspring |
| Gaillard et al. | 2014 | BMI | cardiometabolic profile | maternal and paternal pre-conceptional BMI contribute to an adverse cardiometabolic profile in consecutive generations |
| McCarthy et al. | 2015 | BMI | BMI, WC, triglyceride | significant positive association between paternal BMI and BMI, WC and triglyceride levels of the offspring |
| Santos Ferreira et al. | 2017 | BMI | cardiometabolic profile | paternal BMI could be linked to an detrimental cardiometabolic profile in the later life of the offspring |
| Magnus et al. | 2018 | obesity | childhood-onset type 1 diabetes | paternal pre-conceptional obesity is associated with an increased risk of developing type 1 diabetes in the childhood |
| Labayen et al. | 2010 | BMI | cardiovascular diseases | paternal BMI is positively associated with the offspring’s cardiovascular diseases risk. |
| Veena et al. | 2013 | obesity | cardiovascular risk | paternal obesity showed a positive correlation to obesity and fasting insulin concentrations in offspring;  paternal obesity was associated with an increased childhood BMI and WC, a higher sum of skin folds and an elevated body fat percentage |
| Soubry et al. | 2013 | obesity | DNA methylation patterns of the imprinted Insulin-Like Growth Factor 2 (IGF2) | significant decrease in methylation at the differentially methylated regions (DMRs) of the IGF2 gene among newborns of obese fathers |
| McCowan et al. | 2011 | Birth weight | small for gestational age (SGA) | men who fathered SGA infants showed actually lower birthweights than men who fathered non-SGA infants; risk of delivering an SGA infant was increased by 60% if the father was obese or showed central adiposity |
| Derraik et al. | 2019 | birth weight | large-for-gestational-age (LGA) | likelihood of having an baby which is LGA increased with a higher paternal birth weight and the father being tall |
| Kaati et al. | 2002 | nutrition | cardiovascular deaths, including diabetes mellitus | if the father was exposed to a famine during his slow growth period, the offspring was protected against deaths caused by cardiovascular diseases |
| Li et al. | 2017 | nutrition | hyperglycemia and type 2 diabetes (T2DM) in adulthood | significant increase in the risk of developing hyperglycemia and T2DM in adult offspring of the first generation |
| Penesova et al. | 2010 | diabetes mellitus | insulin secretion | fathers with an onset of diabetes before the age of 35 had leaner children, which further showed a decreased early insulin secretion |
| Silva et al. | 2017 | diabetes mellitus | BMI, triglyceride | link a paternal history of cardiovascular disease, including T2DM, to an increased offspring BMI and elevated triglyceride levels. |
| Wang et al. | 2015 | diabetes mellitus | diabetes mellitus | positive association between parental diabetes and T2DM incidence in offspring |
| Praveen et al. | 2012 | diabetes mellitus | insulin sensitivity and β-cell function | Compared to controls, the offspring with a family history of T2DM showed higher BMI values and higher plasma insulin, C-peptide and proinsulin levels. Moreover, lower insulin sensitivity and β-cell compensation in the offspring was observed |
| Linares Segovia et al. | 2015 | diabetes mellitus | metabolic syndrome | highest BMI values of the offspring were reported in families, in which both parents were diabetic |
| Almari et al. | 2018 | Diabetes mellitus and obesity | prediabetes | parental history of diabetes in addition to obesity in offspring showed an increased appearance of prediabetes |
| Shields et al. | 2006 | insulin resistance | insulin resistance | paternal insulin resistance influences the umbilical cord insulin concentrations in a way which is contributing to the development of a fetal insulin resistance |
| Myklestad et al. | 2012 | BMI | birthweight and glucose levels | relationship between low offspring birthweight and unfavorable glucose levels, increased blood pressure and high BMI values among fathers |
| Hillman et al. | 2013 | insulin resistant, hypertonic and obese | fetal growth-restriction | fathers of offspring with a fetal growth-restriction were more likely to be insulin resistant, hypertonic and obese compared to fathers of normal grown offspring |
| Hyppönen et al. | 2003 | diabetes mellitus | birth weight | relationship between diabetes in fathers and low birth weight of their offspring |
| Moss et al. | 2015 | diabetes mellitus | birth weight | paternal diabetes was associated with a decreased birth weight and lower gestational age of the offspring |
| Lindsay at al. | 2000 | diabetes mellitus | birth weight | development of paternal diabetes can be predicted by the offspring’s birth weight |
| Lauenborg et al. | 2011 | diabetes mellitus | birth weight | paternal history of diabetes is associated with lower birthweight in offspring |
| Zhu et al. | 2018 | age | mortality | U-shaped association between paternal age and mortality rates of children |
| Urhoj et al. | 2014 | age | mortality | positive association between advanced paternal age and the mortality risk of children |
| Zhue et al. | 2005 | age | congenital formations | prevalence of malformations of extremities and syndromes of multiple systems (e.g. Down´s syndrome) increased with advancing paternal age |
| Su et al. | 2015 | age | congenital defects | no clear association between the father’s age and heart defects in offspring |
| Khandwala et al. | 2018 | age | premature birth and birth weight | linked advanced paternal age to an increased risk of premature birth and a low offspring birth weight |
| Marczylo et al. | 2012 | smoking | microRNA (sperm and embryo development) | cigarette smoke induces differential microRNA expression in the spermatozoa of smokers (compared to non-smokers); these altered microRNAs mediate pathways that are essential for sperm and embryo development |
| Pembrey et al. | 2006 | smoking | BMI | transgenerational effect of paternal mid-childhood smoking on offspring BMI at 9 years |
| De Jonge et al. | 2013 | smoking | hypertension | paternal smoking of 15 cigarettes/day or more was associated with an increased risk of hypertension in adult offspring |
| Dior et al. | 2014 | smoking | weight, height, BMI | positive association between maternal and paternal smoking and offspring weight, height and BMI at the age of 17 and a negative association with pulse rates |
| Golding et al. | 2019 | smoking | fat mass | regular paternal cigarette smoking before the age of 11 is strongly associated with an elevated fat mass in adulthood of the respective children |
| Carslake et al. | 2016 | smoking | BMI | linked paternal early-onset smoking (before the age of 11) with higher BMI values in male or female offspring |
| Dougan et al. | 2016 | smoking | obesity | grand-paternal smoking during pregnancy of the grandmother is associated with a higher risk for granddaughters at the age of 12 to be overweight or obese |
| Deng et al. | 2013 | smoking | heart defects | associated with conotruncal heart defects |
| Cresci et al. | 2011 | smoking | heart defects | associated with conotruncal heart defects |
| Robledo et al. | 2015 | persistent organic pollutants | birth size and weight | birth size and weight of the offspring is affected by the pre-conceptional paternal exposure to persistent organic pollutants |
| Lawson et al. | 2004 | exposure to dioxins | adverse pregnancy outcomes | could not provide any evidence that there is an association between paternal exposure to dioxins and adverse pregnancy outcomes |
| Magnusson et al. | 2006 | toxic substances | offspring health | Conflicting results |
| Desrosiers et al. | 2012 | toxic substances | offspring health | Conflicting results |
| **Experimental studies** | | | | |
| Ng et al. | 2010 | high-fat-diet | obesity, insulin resistance | female litter showed adiposity and insulin resistance similar according their fathers |
| Masuyama et al. | 2016 | high-fat-diet | metabolic syndrome | offspring of high-fat diet fed sires showed a metabolic syndrome-like phenomena, which includes risk factors like weight and fat gain, glucose intolerance as well as elevated total triglyceride, decreased adiponectin and increased leptin levels |
| Ornellas et al. | 2015 | obesity | glucose metabolism | offspring showed an impaired glucose metabolism and lipogenesis |
| McPherson et al. | 2015 | obesity | metabolic syndrome | paternal obesity, which was induced by a high-fat diet prior to conception, caused insulin resistance and increased the accumulation of adipose tissue in their female offspring |
| Ng et al. | 2014 | high-fat diet/obesity | adipose tissue | obesity caused insulin resistance and increased the accumulation of adipose tissue in their female offspring |
| Fullston et al. | 2015 | obesity/ high-fat diet | metabolic and sperm disturbances | male mice offspring were affected by a pre-conceptional paternal exposure to a high-fat diet |
| Fullston et al. | 2013 | obesity/ high-fat diet | glucose tolerance, insulin resistance | paternal exposure to a high-fat diet, which caused obesity, induces a specific transgenerational phenotypic constellation of impaired glucose tolerance, insulin resistance in both male and female offspring |
| Fullston et al. | 2012 | high-fat diet | subfertility | high fat diet-induced paternal initiation of subfertility in offspring of two generations of mice. |
| Lecomte et al. | 2017 | high-fat diet | growth | reduced growth hormone as well as IGF2 levels were observed during the first six months of the offspring |
| Krout et al. | 2018 | high-fat diet | T2DM | paternal exercise before conception can reduce the offspring’s risk of developing T2DM, which was induced by the fathers high-fat diet |
| Consitt et al. | 2018 | high-fat diet | insulin sensitivity | paternal high-fat diet enhances skeletal muscle insulin sensitivity as well as whole-body insulin sensitivity in the early life of the offspring |
| Watkins and Sinclair | 2014 | low protein diet | cardiovascular and metabolic homeostasis | adult offspring, which was sired by a low protein diet-fed father, developed a significantly impaired cardiovascular and metabolic homeostasis |
| Carone et al. | 2010 | low protein diet | lipid metabolism | paternal diet affects the gene expression patterns of hundreds of genes of the offspring, especially the signaling pathways of the lipid metabolism |
| Anderson et al. | 2006d | undernutrition | serum glucose | paternal food deprivation caused a consistent decrease in average serum glucose in both male and female offspring + changes in corticosterone and insulin-like growth factor 1 |
| McPherson et al. | 2016 | undernutrition | growth, metabolism | intervention led to dyslipidemia, accumulation of adipose tissue and an altered expression of pancreatic genes in male and female offspring |
| Grasemann et al. | 2012 | hyperglycemia | metabolic phenotype | developed a milder metabolic phenotype, but graver skeletal phenotype |
| Shi et al. | 2017 | hyperglycemia | metabolic profile | adult offspring of hyperglycemic fathers showed a significant weight gain, larger body size and an extensive expansion of adipose tissue resulting in obesity of the offspring |
| Li et al. | 2019 | hyperglycemia | metabolic profile | increased liver weight, elevated plasma total cholesterol, triglyceride and LDL levels and an accumulation of triglycerides in the liver |
| Wei et al. | 2014 | prediabetes | glucose intolerance and insulin resistance | gene expression in the pancreatic islets of offspring of prediabetic fathers was altered and consequently the regulation of the glucose metabolism and insulin signaling pathways showed changes, too. |
| Lane et al. | 2014 | reactive oxygen species (ROS) | glucose | female offspring was particularly affected by the ROS showing an altered body composition and glucose regulation |
| Godschalk et al. | 2018 | genotoxic agent benzo[a]pyrene (B[a]P) | mitochondrial metabolism | down-regulation of mitochondrial proteins and specially in male offspring an additional reduction of mitochondrial DNA copies could be identified |
